# Distinguishing Features Common to Dual Fatal Herpes Simplex Virus Infections That Occur in Both a Pregnant Woman and Her Newborn Infant

**DOI:** 10.3390/v13122542

**Published:** 2021-12-18

**Authors:** Nathan B. Price, Kelly E. Wood

**Affiliations:** 1 Division of Infectious Diseases, Department of Pediatrics, University of Arizona, Tucson, AZ 85719, USA; 2 Newborn Nursery Service and Hospitalist Service, Division of General Pediatrics and Adolescent Medicine, Stead Family Department of Pediatrics, University of Iowa, Iowa, IA 52242, USA; kelly-wood@uiowa.edu

**Keywords:** herpes simplex virus type 1, herpes simplex virus type 2, neonatal herpesvirus infection, HSV UL6, herpesvirus encephalitis, herpesvirus hepatitis, acyclovir, HerpeSelect test, viral sequencing, HELLP syndrome

## Abstract

Deaths from herpes simplex virus type 1 (HSV-1) and herpes simplex virus type 2 (HSV-2) are rare. A major exception is perinatally acquired HSV-1 or HSV-2 infection where the neonatal death rate is substantial. Fatal HSV infection also occurs occasionally in pregnant women. The goal of this review is to enumerate the reports that describe dual deaths of both a pregnant woman and her newborn from a herpesvirus infection. A total of 15 reports were found in the medical literature, of which five described pregnant women with HSV encephalitis and 10 described women with disseminated HSV infection. When the virus was typed, most cases of dual mother/newborn deaths were caused by HSV-2. Of interest, in two situations caused by HSV-1, the pregnant woman probably acquired her primary HSV-1 infection from one of her children and not by sexual transmission. Complete genomic sequencing was performed on one set of HSV-1 isolates collected from mother (blood) and newborn (blood and skin). The mother’s strain and the newborn’s skin strain were 98.9% identical. When the newborn’s two strains were compared, they were 97.4% identical. Only one mother was tested by the HerpeSelect IgG antibody kit. During the nine days of her undiagnosed disseminated infection preceding her death, her serology was negative. In summary, although dual mother/newborn deaths from HSV infection are rare, they continue to be reported as recently as 2017.

## 1. Introduction

**Figure 1:**
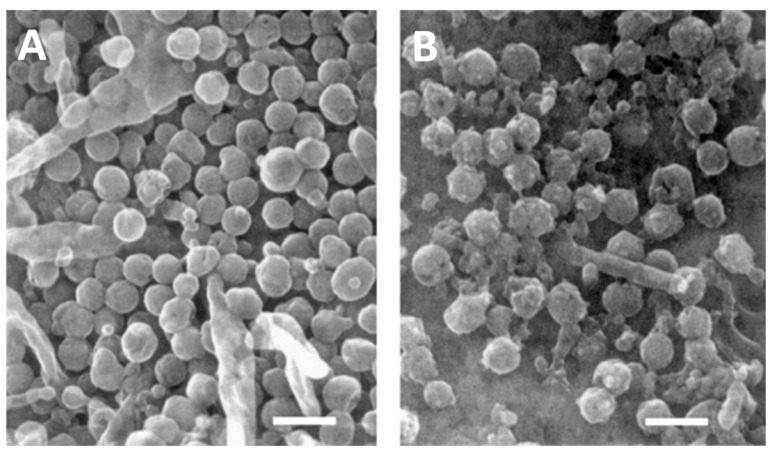
Scanning electron micrographs of herpes viruses. (**A**). HSV-1. (**B**). HSV-2. The appearance of complete enveloped HSV-1 particles is more uniform than HSV-2 particles on the surface of an infected cultured cell. Micrographs obtained from the archives of the University of Iowa Central Microscopy Research Facility. Bar, 400 nanometers.

There are two human herpes simplex viruses, called herpes simplex virus type 1 (HSV-1) and herpes simplex virus type 2 (HSV-2) [1]. Their structures are shown after examination by electron microscopy (EM) (Figure 1). HSV-1 is usually acquired during childhood, as a disease presenting with gingivostomatitis, while HSV-2 is often acquired as a sexually transmitted disease in adolescence and early adulthood [2]. However, over the past 30 years, physicians at several major metropolitan areas, including the University of Washington Medical Center in Seattle, have observed that HSV-1 has become a more common cause of genital disease than HSV-2 [1]. As with all herpesviruses, after primary infection both HSV-1 and HSV-2 enter a latent state within the dorsal root ganglia. Both viruses periodically reactivate throughout the life of an infected individual [3,4,5]. Death from a primary infection or reactivation with either HSV-1 or HSV-2 is exceedingly rare in immunocompetent children and adults [6]. However, when an otherwise healthy woman contracts a primary infection during pregnancy, the likelihood of death rises considerably [7,8]. Furthermore, there is also a concurrent risk that the fetus or newborn infant becomes infected [9]. What is extraordinarily unusual is for both a pregnant woman and her newborn child to die after a primary herpesvirus infection. This rare scenario in medicine is the subject of this review. The goal of the review is to search for further insight into pathogenesis of a herpesvirus infection when both mother and infant die from the same infection.

## 2. Evolution of the Viruses

HSV-1 is an ancient virus that has coevolved with primates throughout 60 million years of evolution in Africa, finally coevolving with ancestors to *Homo sapiens* into contemporary times [10,11]. By contrast, HSV-2, also commonly called genital herpes, evolved around 1 million years ago when a herpes virus from the great ape species bonobo was acquired by *Homo erectus* [12]. The mechanism for this zoonotic spread is likely the same as for human acquisition of HIV-1, namely *Homo erectus* hunted and ate the herpesvirus-infected bonobo for food in East Africa, while contemporary human inhabitants living in West Africa around 100 years ago hunted and ate the retrovirus-infected chimpanzee for food [13]. In other words, Lucy Australopithecus, who lived 3 million years ago in the country now known as Ethiopia would likely have had an HSV-1 infection passed onto her from her parents or siblings, but she could not have had HSV-2 infection, since HSV-2 had not yet arisen [14,15]. However, by the time *Homo sapiens* migrated out of Africa into the Middle East 70,000 years ago, some would have been infected with both HSV-1 and HSV-2. Thus, HSV-1 and HSV-2 infections are found in nearly all human populations distributed around the world. As HSV-1 infected humankind spread into Asia and Europe over past millennia, a sufficient number of polymorphisms have arisen in the genomes, that current strains of HSV-1 can be segregated into Asian and European groupings or clades [16]. There does not appear to be any differences in virulence between the geographic clades of viruses. A similar pattern of geographic genome variation is seen within varicella-zoster virus (VZV) clades [17]. Likewise, there is no difference in virulence between the different geographic VZV clades.

## 3. Past Published Cases of Dual Infection with Dual Deaths

As noted in the Introduction, deaths following HSV infection are rare. After a search of the medical literature, we have found 15 reports of dual deaths of a pregnant woman (Table 1) and her newborn infant (Table 2). We have divided the cases into two groups. The first group includes pregnant women who developed HSV encephalitis (cases 1–5) and the second group includes pregnant women with a disseminated HSV infection (cases 6–15). Vignettes of each case are included below. We recognize that the documentation of HSV infection in some of the earlier cases (especially the newborn) is less than that in the later cases.

### 3.1. Case 1, Minnesota, USA

A 31-year-old woman developed runny nose, headache, hallucinations, confusion, and fever over the course of a week [18]. She was admitted at 7 months of gestation and had no oral HSV lesions on examination. Testing of the cerebrospinal fluid (CSF) showed lymphocytic pleocytosis and an electroencephalogram (EEG) was abnormal. She developed seizures, became comatose and died on the 10th day of hospitalization. Immediately post-mortem, a fetus was delivered by cesarean section (C-section), but the fetus was not viable. Maternal autopsy showed viral cytopathic effect in the brain, and brain tissue culture grew HSV which was not serotyped. Fetal autopsy studies were not done.

### 3.2. Case 2, Massachusetts, USA

A 28-year-old woman developed malaise, slurred speech, amnesia, and unexplained falls over the course of 10 days [19]. After she developed fever and seizures, she was admitted at 28 weeks of gestation. CSF examination showed pleocytosis. She was given empiric antibiotics, but she developed additional seizures and became comatose. She progressively worsened and died 3 weeks after initial onset of symptoms. The day after her hospitalization, a fetus was delivered spontaneously and died within 3 h of birth. Maternal autopsy showed brain edema and necrosis. Culture from the temporal lobe grew HSV, but no typing was done. Infant autopsy showed hyaline membrane disease of prematurity, but no viral cultures were performed.

### 3.3. Case 3, Bogota, Columbia

A 20-year-old woman was admitted with seizures and treated for eclampsia at 28 weeks of gestation [20]. The next day she delivered a dead fetus. Subsequently, the woman developed headache, fever, hallucinations, bizarre behavior and delirium. CSF examination showed lymphocytic pleocytosis. She was started on empiric therapy for tuberculosis. She progressively worsened, became comatose, had worsening seizures and died about 2 weeks after admission. Autopsy showed necrotizing encephalitis involving bilateral temporal lobes with viral inclusion bodies in the neurons and glia suggestive of an HSV infection. No fetal autopsy was done.

### 3.4. Case 4, Bogota, Columbia

A 22-year-old woman was admitted to a psychiatric hospital because of 2 months of bizarre behavior [20]. She was diagnosed with and treated for schizophrenia. At 16 weeks of gestation, she had a spontaneous abortion and was admitted to the hospital for curettage. She then developed hypotension, bradycardia and became unresponsive. She was treated for septic shock but died 3 days after admission. Autopsy showed bitemporal subacute necrotizing encephalitis with viral inclusion bodies in the neurons. Electron microscopy showed abundant herpesvirus-like particles. No fetal autopsy was done. 

### 3.5. Case 5, Bogota, Columbia

A 17-year-old young woman developed bizarre behavior at 24 weeks of gestation [20]. A week later she was admitted to the hospital because of coma. CSF examination showed normal cell count, protein, and glucose. Labor was induced and she delivered a dead fetus. Subsequently, the woman developed fevers, seizures and had an abnormal EEG consistent with temporal lobe involvement. Right temporal lobe biopsy showed edema, necrotizing encephalitis and viral inclusion bodies suggestive of HSV infection. She was treated with steroids but continued to worsen and died 2 weeks after admission. Autopsy findings in the mother were similar to the biopsy findings; no fetal autopsy was done.

### 3.6. Case 6, Texas, USA

A 23-year-old woman developed fever, sore throat, malaise and shortness of breath at 28 weeks of gestation [21]. She was noted to have an erythematous posterior pharynx with ulcerated lesions. Her 2-year-old child at home was recovering from herpes gingivostomatitis at the time. The mother developed worsening respiratory distress and was admitted to the hospital. No fetal heart tones were heard, and the uterus measured the same as it had 2 weeks prior. She had leukocytosis, anemia and proteinuria. She developed hypoxia, hypotension and died within 16 h of hospitalization from cardiopulmonary arrest. Autopsy showed hypopharyngeal ulcers as well as necrosis of the liver and adrenal glands with Cowdrey Type A inclusions. EM showed viral particles consistent with HSV. Tissue cultures grew HSV-1. The fetus appeared normal for gestation on gross examination, but viral cultures were not done.

### 3.7. Case 7, Missouri, USA

A 21-year-old woman at 36 weeks of gestation fell several times at home [22,23]. Over the course of the next 5 days, she developed abdominal pain and fever not responsive to oral antibiotics. She was admitted to the hospital and found to have ruptured membranes with purulent fluid and a cervical lesion with a white pseudomembrane. The mother was treated with antibiotics and the fetus was delivered by C-section within hours of maternal admission. The mother developed multiorgan failure of the liver and kidneys, in addition to encephalopathy. A cervical smear was consistent with HSV infection; a liver biopsy showed necrosis and grew HSV-2. Adenine arabinoside was started on hospital day 9, but she continued to deteriorate and died 12 days after admission. Autopsy showed liver necrosis. The newborn died at 10 days of life and autopsy showed hepatoadrenal necrosis. No cultures were done.

### 3.8. Case 8, Louisiana, USA 

A 33-year-old woman at 24 weeks of gestation developed fever, malaise, nausea, vomiting and abdominal pain over 4 days [7]. She was admitted to the hospital and found to have ulcerative cervicitis. Cultures of lesions later grew HSV, type not noted. She was started on empiric antibiotics, developed hepatitis and underwent exploratory laparotomy; she was found to have many small white nodules on the liver. Antibiotics were started. She continued to deteriorate and had spontaneous contractions; the fetus failed to progress and a stillborn infant was delivered by hysterotomy. Liver biopsy showed necrosis and Cowdrey Type A inclusions. Her condition continued to deteriorate and she developed bleeding, encephalopathy, seizures, respiratory and renal failure; she died on the 14th hospital day. Autopsy showed liver necrosis with Cowdrey type A inclusions and EM inclusions consistent with herpesvirus. She also had pulmonary embolism, cerebral edema and cerebellar tonsillar herniation. Tissue cultures were negative. No autopsy of the fetus was done.

### 3.9. Case 9, Michigan, USA

An 18-year-old woman at 34 weeks of gestation developed fever, malaise and otalgia over 4 days [24,25]. She had no mucocutaneous HSV lesions on exam. She was admitted with hepatitis and started on empiric antibiotics. Her hepatitis worsened and she developed coagulopathy. The infant was delivered by C-section 5 days after admission. The mother developed seizures and died 4 days after the infant was delivered. Autopsy showed liver necrosis. Liver, rectal and throat cultures grew HSV, type not specified. HSV antibodies were negative on admission and positive on hospital day 8. The infant developed hyaline membrane disease of prematurity and intraventricular hemorrhage and died 2 days after delivery. The infant’s culture results for HSV were negative.

### 3.10. Case 10, Hamburg, Germany

A 20-year-old woman was treated for 2 weeks with antibiotics for pyelonephritis at one hospital l [26]. She developed hepatitis, coagulopathy and was transferred to a different hospital at 34 weeks of gestation. Liver biopsy done at the prior facility showed necrosis and she was started on acyclovir. The mother’s condition worsened and the fetus showed signs of distress; a C-section was done 24 h after transfer. The mother had some improvement for about 12 h, then developed seizures, encephalopathy and respiratory failure. She died 4 days after transfer. Autopsy showed pyelitis of the kidney and liver necrosis. Culture from the liver grew HSV-2. The infant was treated with acyclovir but died of encephalitis at 14 days of age. Intrathecal antibodies were positive for HSV-2.

### 3.11. Case 11, South Carolina, USA

A 30-year-old woman at 31 weeks of gestation was admitted with a 2-day history of fever, cough and abdominal tenderness [27]. She was started on antibiotics for pyelonephritis. She developed hypoxia, pulmonary infiltrates, coagulopathy and hepatitis. Fetal distress was noted and the infant was delivered by C-section on hospital day 5. One of four maternal blood cultures grew Group B Streptococcus. She developed respiratory and renal failure and died on hospital day 9. Autopsy showed liver necrosis, and immunostaining showed Cowdry type A inclusions positive for HSV. Liver tissue cultures grew HSV 2. Inclusions were also seen in lung and spleen tissue. The infant developed hyaline membrane disease and was started on acyclovir on day 6 after the possibility of HSV infection of the mother was raised. Despite this therapy, the infant died at 11 days of life. Cultures from the infant’s blood, urine, throat and CSF grew HSV-2. Autopsy was not done.

### 3.12. Case 12, Texas, USA

A 21-year-old woman at 12 weeks of gestation had fever, myalgia, urinary frequency and urgency for 3 days not responsive to nitrofurantoin [8]. She was admitted and started on empiric antibiotics. She had no mucocutaneous lesions. She developed cough, respiratory distress and lung infiltrates on chest film, followed by hepatitis and coagulopathy. She worsened and died 8 days after admission. Autopsy of the woman showed necrotizing bronchopneumonia with Cowdry type A inclusions in the lung that stained positive for HSV. Liver necrosis was seen with positive HSV staining, and cultures grew HSV 2. Uterine necrosis was seen with few cells suspicious for viral inclusions, but uterine tissue cultures were negative for HSV. A fetal autopsy was not performed.

### 3.13. Case 13, California, USA

A 21-year-old woman at 27 weeks of gestation presented with fever and vaginal discharge [28]. She was found to have a right lower lobe pneumonia and leukopenia and did not respond to 4 days of empiric antibiotics. She was transferred to a higher-level medical facility. She did not have any mucocutaneous lesions. She developed hepatitis, coagulopathy and within 24 h of transfer she developed hypotension and respiratory failure. Fetus had appeared healthy, but labor was induced due to risk of ongoing sepsis to the fetus. C-section was not attempted due to maternal condition and the fetus was delivered stillborn 9 h later. The mother’s condition continued to worsen, and she developed skin vesicles on her thigh, forearm and back. She was started on acyclovir and cultures of the lesions grew HSV-2. She developed anasarca and then died after repeated episodes of ventricular tachycardia 18 days after admission. Autopsy showed liver and lung necrosis with immunohistochemical staining positive for HSV. Autopsy of the fetus was not done.

### 3.14. Case 14, Ohio, USA

An 18-year-old woman at 26 weeks of gestation developed shortness of breath and fever [29]. She was admitted and found to have hepatitis and coagulopathy. A fetus was delivered by C-section and mother continued to deteriorate; she was transferred for liver transplant evaluation. She developed renal and respiratory failure and was started on acyclovir on hospital day 3. She died of multiorgan failure on hospital day 9. A blood HSV-2 polymerase chain reaction (PCR) test and a herpes IgM test were positive. The newborn died of HSV-2 sepsis at 4 days of life.

### 3.15. Case 15, Iowa, USA

This dual-case where both the pregnant mother and her infant died from HSV-1 infection has been described in two reports [30,31]. These are summarized in the section below.

## 4. A Recent Case of Fatal Dual Infection

**Figure 2:**
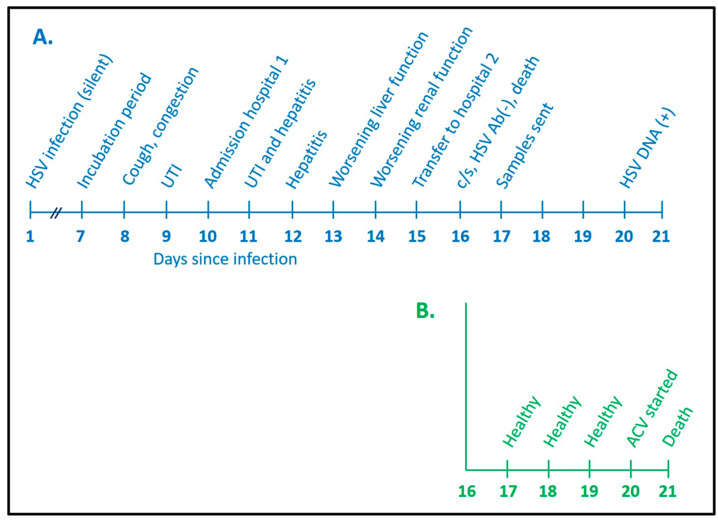
Timeline of hospitalization of pregnant woman and her newborn infant. For this timeline, a typical incubation period for primary HSV-1 infection was considered to be 7 days [6]. The diagram documents a day-by-day rapid progression to death of a pregnant woman (**A**, blue) and her newborn infant (**B**, green) after disseminated HSV-1 infection in the woman. UTI, urinary tract infection; c/s, cesarean section; ACV, acyclovir.

The mother in Case 15 was a 41-year-old female in the 29th week of gestation during her sixth pregnancy [30]. She had two living children and three prior miscarriages. She was admitted to a hospital because of a 2-day history of fever and cough (Figure 2). During an earlier clinic visit, she had been diagnosed with a urinary tract infection. After an Infectious Disease consultation, she was treated with ceftriaxone and oseltamivir. Her liver function tests on admission included aspartate aminotransferase (AST) 90 units and alanine aminotransferase (ALT) 61 units, and her creatinine was 0.7 mg/dL. Within 3 days, her AST and ALT levels were 5652 and 1559 and her creatinine was 3.2. Her platelet count had fallen from 172,000 to 139,000/mm^3^. She became disoriented. The main diagnoses that were considered included HELLP syndrome (hemolysis, elevated liver enzymes and lowered platelets) and acute fatty liver of pregnancy [32]. She was transferred on an emergent basis to a referral hospital, where a C-section was performed around 2 AM. An order was placed for a HerpeSelect IgG antibody test (Focus Diagnostics, Cypress, CA, USA) after the C-section and the results showed no detectable antibody to either HSV-1 or HSV-2. Her hepatic function and renal function continued to worsen throughout the day, and she died around 11 PM.

A complete autopsy was performed on the following day. Numerous additional samples were collected and sent, mainly to outside diagnostic facilities. These tests included several PCR assays. The results were returned 3 days later. A PCR assay for HSV-1 was positive in a blood sample. Subsequent immunohistochemical analysis of the liver and adrenal glands showed multiple foci of infection by HSV-1, but no foci of HSV-1 infection were found in the kidney. In Figure 2, we have placed the time of HSV-1 infection for the mother as one week before she began to develop symptoms of illness (incubation period); she died 16 days after contracting the infection.

The transfer note from the obstetrics staff to the neonatology staff cited severe preeclampsia as the reason for the emergency C-section [31]. The newborn underwent a physical examination by neonatologists shortly after the C-section; although premature, the examination and weight (1.6 kg) were normal for 30 weeks of gestation (Figure 2). The blood indices and ALT test were also normal. A similar normal examination was recorded during the first 3 days of life. However, when the deceased mother’s positive HSV-1 PCR results were returned on day of life 4, the infant had begun having apneic spells; he was immediately placed on IV acyclovir (60 mg/kg/day). The neonate died on day 5 of life of disseminated HSV-1 infection. A blood PCR test collected on day 4 subsequently was found to be positive for HSV-1. The infant never developed a skin rash. An autopsy was not performed on the neonate.

Since the newborn had a normal physical examination and a normal ALT test on the first day of life, we speculate that the newborn may have contracted HSV-1 infection during the emergent C-section. An alternative possibility is that the fetus was infected via placental transfer a few days or a few hours before the C-section. Overall, however, intrauterine HSV infection is an uncommon event [1,9].

## 5. Pathogenesis of Fatal Herpes Infection in Infected Pregnant Women

After a review of the 15 reports, we have delineated two very different modes of pathogenesis leading to death of both pregnant mother and newborn by HSV infection. The first is HSV encephalitis in the pregnant women. Several reports about the pathogenesis of HSV encephalitis are in the medical literature and this illness will not be further discussed [33,34]. The second is a disseminated HSV infection in the mother, sometimes called herpes sepsis [35]. Furthermore, because of this review, we in turn have distinguished two forms of disseminated HSV infection in the pregnant woman. With regard to HSV-1, in both reported cases, we speculate that the mother acquired a primary HSV-1 infection from one of her young children with or without gingivostomatis, probably through exchange of oral secretions laden with HSV-1. Mother #15 was the oldest mother in Table 1 to die from disseminated herpes. She was a homemaker who also ran a private childcare center in her home. We speculate that she contracted a primary HSV-1 infection from either one of her own children or from one of the children in childcare. This event probably occurred around 2 weeks before she was admitted to hospital. Thus, this case closely resembles case #6, where the mother probably acquired HSV-1 from her child. This mode of HSV-1 transmission from an HSV-1 seropositive child to an HSV-1-seronegative mother is strikingly similar to a common mode of transmission when a CMV-seronegative pregnant women acquires a primary CMV infection from her CMV-seropositive child and subsequently delivers a newborn with a congenital CMV infection [36]. Since the seroprevalence of HSV-1 antibody in American adolescents is only 31% [37], a majority of young pregnant women are at risk of a primary HSV-1 infection. 

**Figure 3:**
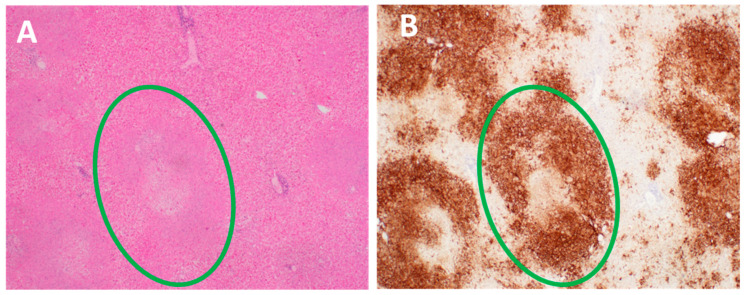
Pathology in liver infected with HSV. Inspection of the surface of the liver demonstrated numerous nodules (2–5 mm in diameter). (**A**). Hematoxylin and eosin stain of an HSV-infected liver section. Viral cytopathology is not easily distinguished by routine staining (green circle). (**B**). Immunohistochemistry. Viral cytopathology is easily distinguished after special immunostaining of an HSV-infected liver with anti-HSV antibody. The dark brown color indicates necrotic viral foci (green circle). Micrographs obtained from the archives of the University of Iowa Department of Pathology. Magnification, 20×.

A second possible mode of HSV-1 acquisition by mother #15 was sexual; under this scenario, her early UTI may have been a misdiagnosed HSV-1 primary genital infection. With regard to HSV-2, all the dual deaths listed in Table 1 and Table 2 followed dissemination from a primary or recurrent genital infection [38,39]. One of the maternal organs most often damaged by disseminated HSV infection is the liver (Figure 3). Liver necrosis caused by extensive HSV-2 replication was documented in all the autopsies from cases 6 to 15 (Table 1).

## 6. Herpesvirus Diagnostic Testing

For diagnosis of a disseminated herpesvirus infection, a PCR-based HSV test of blood is preferred; for diagnosis of herpesvirus encephalitis, a PCR-based HSV test of cerebrospinal fluid is preferred, and for diagnosis of a herpesvirus genital infection, a PCR-based HSV test of swabs collected from the genital area is preferred [1]. Herpesvirus serology is not recommended because the results can be confusing. This report illustrates that a pregnant woman can contract a primary HSV-1 infection, develop HSV-1 viremia, transmit the virus to her fetus, undergo C-section, and then die from an HSV-1 sepsis syndrome before her immune system produces HSV-1 specific IgG antibody detectable by the HerpeSelect Test. This conclusion was not readily apparent from the medical literature, where older reviews of herpesvirus infection during pregnancy include tables where many of the included maternal cases have detectable HSV-1 antibody [8]. Our dual case report indicates that there may be a delay in virus specific antibody responses in pregnant women when tested by the HerpeSelect assay. The HerpeSelect test can discriminate HSV-1 and HSV-2 antibodies because the test contains only one viral protein as antigen (gG protein); the gG protein between HSV-1 and HSV-2 does not share epitopes. 

**Figure 4:**
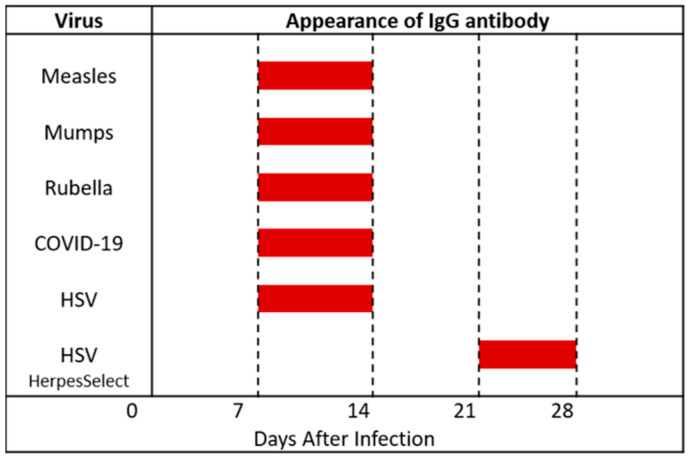
Timeline of appearance of antiviral IgG antibodies in human serum samples. The table shows the timeline by weeks post-infection when specific human IgG antibodies against several common viral pathogens usually are detectable (red rectangles). Data from references [40,41,42,43].

Virologists have already titrated sera collected periodically from patients with primary HSV-1 infection and patients with primary HSV-2 infection [40]. The ages of the HSV-1 patients ranged from 2 to 27 years, while the ages of the HSV-2 patients ranged from 29 to 43 years. HSV-1 specific IgG antibody was detected first between 5 to 10 days, while HSV-2 specific IgG antibody was detected by 15 days. Thus, IgG antibody to HSV-1 or HSV-2 was detectable in assays that used whole virus antigen by 13–15 days after primary infection, rather than just the HSV gG protein. This age range also fits perfectly with testing for appearance of IgG antibody to other viruses, such as measles, mumps, rubella and COVID-19 (Figure 4) [41,42].

The sensitivity of the HerpeSelect test has been assessed in over 100 patients [43]. In patients with culture-documented first episodes of genital herpes, the median time from onset of symptoms to seroconversion by HerpeSelect ELISA was 25 days for patients with primary genital HSV-1 infection and 21 days for those with primary HSV-2 infection (Figure 4). Thus, the HerpeSelect IgG test appears highly specific but less sensitive than older HSV testing methods for detection of the earliest IgG antibodies, which are directed against HSV-1 proteins called gB and Gd [44].

## 7. Genomic Analyses of the HSV-1 Strains

Extensive sequencing was performed on the viral isolates collected from dual case 15. HSV-1 was isolated from the blood of the mother and from the skin (around the mouth and nose) and blood of the newborn. Complete genomic sequencing was performed on each of the three strains at the PennState Huck Institute of the Life Sciences [31]. The entire HSV-1 genome contains 152 kilobases and encodes over 80 genes. The mother’s strain and the infant’s skin strain were 98.9% identical and the mother’s strain and the infant’s blood strain were 96.8% identical. When the baby’s two strains were compared, they were 97.4% identical. The majority of the genetic variations were found in the noncoding regions of the genes and in intergenic regions.

Minor variants were found in the open reading frames within each of the HSV-1 strains. Minor variants are alternative alleles that exist in extremely low frequency in any population of a single virus strain. A total of 39 minor variants were detected in the maternal strain, 42 minor variants in the neonate’s skin strain and 28 minor variants in the infant’s blood strain [31]. By identification of these minor variants, it was apparent that some minor variants in the mother’s HSV-1 strain were found in both of the infant’s two strains. Thus, there did not appear to be a bottleneck event during transmission of virus from pregnant mother to infant, presumably at the time of the emergency C-section. However, of more interest, there were 23 minor variants in the infant’s two HSV-1 strains that were not present in the maternal strain [31]. This result indicated that HSV-1 genetic mutations had occurred in the infant’s HSV- population within only 6 days of life.

**Figure 5:**
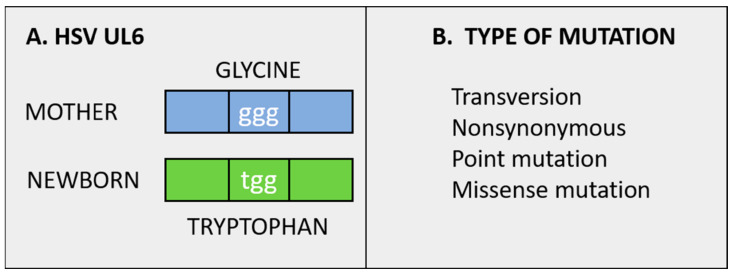
Mutation in codon 486 of HSV UL6 portal protein. (**A**). Diagram of UL6. (**B**). Names of the UL6 mutation. This missense mutation in one open reading frame of the HSV genome was a minor variant detected in the blood of an HSV-infected newborn infant who lived for only 6 days. The variant arose in the infant because it was not detected in the sequencing of the maternal HSV-1 strain.

The single most important nonsynonymous minor variant that occurred in theinfant’s two HSV1 strains was found in an HSV-1 gene known as unique-long 6 (UL6) (Figure 5). The HSV1 structural protein is called the portal protein because it is attached to the viral capsid, where it facilitates entry and packaging of newly synthesized viral DNA genomes from the nucleus into the capsid itself [45]. After the viral DNA is packaged within a capsid, the capsid exits the nucleus and enters the cytoplasm, where the capsid undergoes envelopment before finally being released from the infected cell. Since no experiments have yet been performed with the UL6 variant, we do not know if the mutation documented in the HSV-1 portal protein of the infant hinders or accelerates encapsidation of viral genomes. The GenBank accession numbers for the maternal strain, infant’s skin strain, and infant’s blood strain are listed, respectively: MK 952185, MK 952183, and MK 952184. 

## 8. Conclusions

Our survey of the literature has uncovered 15 reports of dual mother/newborn deaths from HSV infection, more than found in other reviews (Table 1 and Table 2). The fact that HSV encephalitis may occur more commonly in pregnant women has not been widely discussed. Nevertheless, dual deaths following maternal HSV encephalitis seem to be a disease that has largely disappeared, probably due to the frequent use of intravenous acyclovir treatment in any person suspected of having HSV encephalitis [46].

In sharp contrast, dual mother/newborn deaths following primary HSV-1 infections in a pregnant woman are still being reported. As shown in case #15, the symptoms of a primary HSV-1 infection in the mother can closely resemble other noninfectious illnesses of pregnant women. A negative HerpeSelect IgG titer may be a clue that the pregnant woman potentially has a primary HSV-1 infection. Obviously an HSV-1 DNA PCR test must be performed on a throat swab and a blood sample to confirm this diagnosis. We speculate that both of our HSV-1 cases may have been caused by HSV-1 transmission from a child to a pregnant mother. There were no documented dual deaths from an HSV-1 primary maternal genital infection in the above 15 reports. Whether the six dual-mother/newborn deaths from a documented disseminated HSV-2 infection were caused by a primary HSV-2 infection or the first clinical presentation of a previously acquired but asymptomatic HSV-2 infection cannot be determined by the limited virological testing preformed on the six pregnant women in the above case reports. 

Dual deaths of a pregnant woman and her newborn from disseminated HSV infection remain a rare event, with only 10 cases reported in the USA since 1966. In contrast, 68 newborns diagnosed with perinatal HSV infection died in 2017 in the USA [47,48]. This number represents a doubling of the rate of neonatal deaths due to HSV infection in 1995. Based on this review, we reach a counter-intuitive conclusion that the more sick the mother is with disseminated HSV infection (in the absence of rash), the greater the likelihood of an incorrect diagnosis, such as HELLP syndrome or acute fatty liver of pregnancy [32]. There are two pathways by which to reduce the number of deaths from herpesvirus infections: (i) upgrade clinical microbiology laboratories at community hospitals or (ii) develop a herpesvirus vaccine. Currently many community hospitals do not have the technology to perform same-day PCR analyses on a variety of clinical samples. On the other hand, there is considerable interest in the development of a vaccine against both HSV-1 and HSV-2 [49]. Promising experimental data about immunization within a mouse model system have shown that HSV-2 vaccination of a pregnant mouse can prevent HSV-2 infection in her newborn pups [50].

## Figures and Tables

**Table 1:** Fatal Herpes in Mothers.

Case #	Year	Age	Serotype	Findings at Death	Day of Death	Reference
1	1966	31	unk ^a^	Encephalitis	10	[18]
2	1975	28	unk	Encephalitis	11	[19]
3	1979	20	unk	Encephalitis	14	[20]
4	1979	22	unk	Encephalitis	3	[20]
5	1979	17	unk	Encephalitis	14	[20]
6	1974	23	1	Liver necrosis	0.5	[21]
7	1980	21	2	Liver necrosis	12	[22,23] ^b^
8	1982	33	unk	Liver necrosis	14	[7]
9	1985	18	unk	Liver necrosis	9	[24,25] ^b^
10	1992	20	2	Liver necrosis	4	[26]
11	1996	30	2	Liver necrosis	9	[27]
12	1996	21	2	Liver necrosis	8	[8]
13	2002	27	2	Liver necrosis	18	[28]
14	2017	18	2	Liver necrosis	9	[29]
15	2017	41	1	Liver necrosis	7	[30,31] ^b^

Note: ^a^, unk, serotype not known; ^b^, same case reported by two different author groups.

**Table 2:** Fatal Herpes in Fetuses and Newborns.

Case #	Year	Serotype	Maternal Infection	GA (Weeks)	Age at Death (Days)	Antiviral
1	1966	Unk	encephalitis	28	IUFD	No
2	1975	Unk	encephalitis	28	0	No
3	1979	Unk	encephalitis	28	0	No
4	1979	Unk	encephalitis	16	0	No
5	1979	Unk	encephalitis	24	0	No
6	1974	1	disseminated	28	IUFD	No
7	1980	2	disseminated	36	10	No
8	1982	Unk	disseminated	25	IUFD	No
9	1985	Unk	disseminated	34	2	No
10	1992	2	disseminated	34	14	Yes
11	1996	2	disseminated	32	11	Yes
12	1996	2	disseminated	13	IUFD	No
13	2002	2	disseminated	27	1	No
14	2017	2	disseminated	26	4	No
15	2017	1	disseminated	30	5	Yes

Abbreviations: GA: gestational age; Unk: serotesting not performed; IUFD: Intrauterine fetal death.

## References

[1] Melvin A.J., Mohan K.M., Vora S.B., Selke S., Sullivan E., Wald A. (2021). Neonatal herpes simplex virus infection: Epidemiology and outcomes in the modern era. J. Pediatric Infect. Dis. Soc..

[2] Nahmias A.J., Roizman B. (1973). Infection with herpes-simplex viruses 1 and 2. N. Engl. J. Med..

[3] Stevens J.G. (1975). Latent herpes simplex virus and the nervous system. Curr. Top. Microbiol. Immunol..

[4] Bloom D.C. (2016). Alphaherpesvirus Latency: A Dynamic State of Transcription and Reactivation. Adv. Virus Res..

[5] Sawtell N.M., Thompson R.L. (2021). Alphaherpesvirus Latency and Reactivation with a Focus on Herpes Simplex Virus. Curr. Issues Mol. Biol..

[6] Juretic M. (1966). Natural history of herpetic infection. Helv. Paediatr. Acta.

[7] Wertheim R.A., Brooks B.J., Rodriguez F.H., Lesesne H.R., Jennette J.C. (1983). Fatal herpetic hepatitis in pregnancy. Obstet. Gynecol..

[8] Young E.J., Chafizadeh E., Oliveira V.L., Genta R.M. (1996). Disseminated herpesvirus infection during pregnancy. Clin. Infect. Dis..

[9] Kimberlin D.W. (2005). Herpes simplex virus infections in neonates and early childhood. Semin. Pediatr. Infect. Dis..

[10] McGeoch D.J., Davison A.J., Domingo E., Webster R., Holland J. (1999). The molecular evolutionary history of the herpesviruses. Origin and Evolution of Viruses.

[11] Davison A.J. (2010). Herpesvirus systematics. Vet. Microbiol..

[12] Wertheim J.O., Hostager R., Ryu D., Merkel K., Angedakin S., Arandjelovic M., Ayimisin E.A., Babweteera F., Bessone M., Brun-Jeffery K.J. (2021). Discovery of Novel Herpes Simplexviruses in Wild Gorillas, Bonobos, and Chimpanzees Supports Zoonotic Origin of HSV-2. Mol. Biol. Evol..

[13] Sharp P.M., Hahn B.H. (2011). Origins of HIV and the AIDS pandemic. Cold Spring Harb. Perspect. Med..

[14] Johanson D.C. (2009). Lucy’s Legacy: The Quest for Human Origins.

[15] Grose C., Johanson D.C. (2016). Transmission of Cytomegalovirus, Epstein-Barr Virus, and Herpes Simplex Virus Infections: From the Lucy Australopithecus Epoch to Modern-Day Netherlands. J. Pediatr..

[16] Szpara M.L., Gatherer D., Ochoa A., Greenbaum B., Dolan A., Bowden R.J., Enquist L.W., Legendre M., Davison A.J. (2014). Evolution and diversity in human herpes simplex virus genomes. J. Virol..

[17] Grose C. (2012). Pangaea and the Out-of-Africa Model of Varicella-Zoster Virus Evolution and Phylogeography. J. Virol..

[18] Rawls W.E., Dyck P.J., Klass D.W., Greer H.D., Herrmann E.C. (1966). Encephalitis associated with herpes simplex virus. Ann. Intern. Med..

[19] Jewett J.F. (1975). Committee on Maternal Welfare Herpes simplex encephalitis. N. Engl. J. Med..

[20] Roman-Campos G., Navarro de Roman L.I., Toro G., Vergara I. (1979). Herpes encephalitis in pregnancy. Am. J. Obstet. Gynecol..

[21] Goyette R.E., Donowho E.M., Hieger L.R., Plunkett G.D. (1974). Fulminant herpesvirus hominis hepatitis during pregnancy. Obstet. Gynecol..

[22] Kobbermann T., Clark L., Griffin W.T. (1980). Maternal death secondary to disseminated herpesvirus hominis. Am. J. Obstet. Gynecol..

[23] Hamory B.H., Luger A., Kobberman T. (1981). Herpesvirus hominis hepatitis of mother and newborn infant. South. Med. J..

[24] Goyert G.L., Bottoms S.F., Sokol R.J. (1985). Anicteric presentation of fatal herpetic hepatitis in pregnancy. Obstet. Gynecol..

[25] Jacques S.M., Qureshi F. (1992). Herpes simplex virus hepatitis in pregnancy: A clinicopathologic study of three cases. Hum. Pathol..

[26] Wolf H., Kuhler O., Henke P., Klose G. (1992). Liver dystrophy in disseminated herpes simplex infection in pregnancy. Geburtshilfe Frauenheilkd..

[27] Gelven P.L., Gruber K.K., Swiger F.K., Cina S.J., Harley R.A. (1996). Fatal disseminated herpes simplex in pregnancy with maternal and neonatal death. South. Med. J..

[28] Frederick D.M., Bland D., Gollin Y. (2002). Fatal disseminated herpes simplex virus infection in a previously healthy pregnant woman. A case report. J. Reprod. Med..

[29] Natu A., Iuppa G., Packer C.D. (2017). Herpes Simplex Virus Hepatitis: A Presentation of Multi-Institutional Cases to Promote Early Diagnosis and Management of the Disease. Case Rep. Hepatol..

[30] Masadeh M., Shen H., Lee Y., Gunderson A., Brown K., Bellizzi A., Tanaka T. (2017). A fatal case of herpes simplex virus hepatitis in a pregnant patient. Intractable Rare Dis. Res..

[31] Shipley M.M., Renner D.W., Pandey U., Ford B., Bloom D.C., Grose C., Szpara M.L. (2019). Personalized viral genomic investigation of herpes simplex virus 1 perinatal viremic transmission with dual fatality. Cold Spring Harb. Mol. Case Stud..

[32] Rath W., Tsikouras P., Stelzl P. (2020). HELLP Syndrome or Acute Fatty Liver of Pregnancy: A Differential Diagnostic Challenge: Common Features and Differences. Geburtshilfe Frauenheilkd..

[33] Whitley R.J. (2006). Herpes simplex encephalitis: Adolescents and adults. Antiviral. Res..

[34] Kennedy P.G., Steiner I. (2013). Recent issues in herpes simplex encephalitis. J. Neurovirol..

[35] Young E.J., Killam A.P., Greene J.F. (1976). Disseminated herpesvirus infection. Association with primary genital herpes in pregnancy. JAMA.

[36] Pass R.F., Hutto S.C., Reynolds D.W., Polhill R.B. (1984). Increased frequency of cytomegalovirus infection in children in group day care. Pediatrics.

[37] Xu F., Lee F.K., Morrow R.A., Sternberg M.R., Luther K.E., Dubin G., Markowitz L.E. (2007). Seroprevalence of herpes simplex virus type 1 in children in the United States. J. Pediatr..

[38] Vontver L.A., Hickok D.E., Brown Z., Reid L., Corey L. (1982). Recurrent genital herpes simplex virus infection in pregnancy: Infant outcome and frequency of asymptomatic recurrences. Am. J. Obstet. Gynecol..

[39] Corey L., Wald A. (2009). Maternal and neonatal herpes simplex virus infections. N. Engl. J. Med..

[40] Kurtz J.B. (1974). Specific IgG and IgM antibody responses in herpes-simplex-virus infections. J. Med. Microbiol..

[41] Evans A.S. (1976). Viral Infections of Humans.

[42] Mulligan M.J., Lyke K.E., Kitchin N., Absalon J., Gurtman A., Lockhart S., Neuzil K., Raabe V., Bailey R., Swanson K.A. (2020). Phase I/II study of COVID-19 RNA vaccine BNT162b1 in adults. Nature.

[43] Ashley-Morrow R., Krantz E., Wald A. (2003). Time course of seroconversion by HerpeSelect ELISA after acquisition of genital herpes simplex virus type 1 (HSV-1) or HSV-2. Sex. Transm. Dis..

[44] Kuhn J.E., Dunkler G., Munk K., Braun R.W. (1987). Analysis of the IgM and IgG antibody response against herpes simplex virus type 1 (HSV-1) structural and nonstructural proteins. J Med Virol.

[45] Newcomb W.W., Juhas R.M., Thomsen D.R., Homa F.L., Burch A.D., Weller S.K., Brown J.C. (2001). The UL6 gene product forms the portal for entry of DNA into the herpes simplex virus capsid. J. Virol..

[46] Dodd K.C., Michael B.D., Ziso B., Williams B., Borrow R., Krishnan A., Solomon T. (2015). Herpes simplex virus encephalitis in pregnancy—A case report and review of reported patients in the literature. BMC Res. Notes.

[47] Donda K., Sharma M., Amponsah J.K., Bhatt P., Okaikoi M., Chaudhari R., Dapaah-Siakwan F. (2019). Trends in the incidence, mortality, and cost of neonatal herpes simplex virus hospitalizations in the United States from 2003 to 2014. J. Perinatol..

[48] Slutsker J.S., Schillinger J.A. (2021). Assessing the Burden of Infant Deaths Due to Herpes Simplex Virus, Human Immunodeficiency Virus, and Congenital Syphilis: United States, 1995 to 2017. Sex. Transm. Dis..

[49] Johnston C., Gottlieb S.L., Wald A. (2016). Status of vaccine research and development of vaccines for herpes simplex virus. Vaccine.

[50] Patel C.D., Backes I.M., Taylor S.A., Jiang Y., Marchant A., Pesola J.M., Coen D.M., Knipe D.M., Ackerman M.E., Leib D.A. (2019). Maternal immunization confers protection against neonatal herpes simplex mortality and behavioral morbidity. Sci. Transl. Med..

